# Strengthening Management, Community Engagement, and Sustainability of the Subnational Response to Accelerate Malaria Elimination in Namibia

**DOI:** 10.4269/ajtmh.21-1195

**Published:** 2022-04-11

**Authors:** Amanda Marr Chung, Eliza Love, Julie Neidel, Idah Mendai, Sakeus Nairenge, Lesley-Anne van Wyk, Sara Rossi, Erika Larson, Peter Case, Jonathan Gosling, Greyling Viljoen, Macdonald Hove, Bruce Agins, Jerobeam Hamunyela, Roland Gosling

**Affiliations:** ^1^ Institute for Global Health Sciences, University of California–San Francisco, California;; ^2^ Ministry of Health and Social Services, Namibia;; ^3^ Independent Consultant;; ^4^ Bristol Business School, University of West of England, United Kingdom;; ^5^ College of Business, Law & Governance, James Cook University, Australia;; ^6^ Business School, University of Exeter, United Kingdom;; ^7^ Department of Disease Control, London School of Hygiene and Tropical Medicine, United Kingdom

## Abstract

Leadership and management skills are critical for health programs to deliver high-quality interventions in complex systems. In malaria-eliminating countries, national and subnational health teams are reorienting strategies to address focal transmission while preventing new cases and adapting to decentralization and declines in external financing. A capacity-strengthening program in two regions in Namibia helped malaria program implementers identify and address key operational, political, and financial challenges. The program focused on developing skills and techniques in problem-solving and teamwork, engaging decision-makers, and using financial evidence to prioritize domestic resources for malaria through participatory approaches. Results of the program included an observed 40% increase in malaria case reporting, 32% increase in reporting and tracing of imported malaria cases, 10% increase in malaria case management, integration of malaria activities into local operational plans, and an increase in subnational resources for malaria teams. To promote program sustainability beyond the implementation period, key program aspects were institutionalized into existing health system structures, program staff were trained in change leadership, and participants integrated the skills and approaches into their professional roles. A capacity-strengthening program with joint focus on leadership, management, and advocacy has potential for application to other health issues and geographies.

## INTRODUCTION

National health programs with the goal of eliminating malaria must contend with the difficult, “last mile” cases. Once malaria transmission has been reduced to low levels, residual transmission requires a more nuanced approach beyond blanket coverage of indoor residual spraying (IRS) and long-lasting insecticidal nets (LLIN).^1^^,^^2^ Subnational health teams need to assume increased responsibility for the malaria response as malaria transmission becomes more focal and as interventions are aimed at specific populations at high risk of malaria.^3^ In resource-constrained settings, the associated operational challenges often result in gaps in identifying and treating malaria infections.^4^ However, these operational challenges can be addressed by strengthening subnational leadership and management capacity and by deploying evidence-based interventions and resources that aim to optimize how a malaria program organizes itself, engages the community, and makes data-informed decisions.^5^^–^^7^ A critical training gap in leadership and management for malaria elimination was identified by Wirth et al.^8^

In addition to operational and technical challenges, malaria elimination programs are often faced with a decline in external funding and lower political prioritization of malaria that can result in a diversion of resources to other health and development challenges that are perceived by decision-makers as more urgent. These financial and political challenges must be met with commensurate advocacy and domestic resource mobilization to sustain the malaria elimination response. In Namibia, external funding for the National Vector-borne Diseases Control Program (NVDCP) has decreased. More specifically, from 2014–2016 to 2020–2022, there was a 50% reduction in the allocation from the Global Fund to Fight AIDS, Tuberculosis and Malaria.^9^ Domestic spending on malaria remains consistently low, at less than 1% of total health expenditure, and significant funding gaps prohibit the full implementation of Namibia’s malaria strategic plan.^10^^,^^11^

Between 2019 and 2021, the Namibia NVDCP, the Kavango East and Kavango West Regional Health Directorate, and two district health teams within these regions (Nankudu and Rundu), collaborated with partners from the authors’ institutes and the Center for Economic Governance and Accountability in Africa (CEGAA) to strengthen subnational leadership, management, and advocacy capacities. This catalytic program used two complementary approaches that aimed to achieve the following:

1. Resolve district health team management and operational barriers and strengthen leadership skills by improving teamwork, decision-making, and planning. This objective involved use of the Leadership and Engagement for Improved Accountability and Delivery of Services (LEAD) Framework for malaria management strengthening.^12^
2. Mobilize regional political commitment and domestic resources by building advocacy and partnership skills and improving understanding of financial flows and public financial management among regional health teams. This objective used the Malaria Budget Advocacy (MBA) Framework.^13^

Although the underlying frameworks have been deployed individually in other countries in Asia Pacific and southern Africa, this was the first time they were implemented in the same country.^14^ Joint implementation of these approaches offers lessons in improving leadership and management of malaria elimination programs by subnational health teams in the context of reduced donor financing, stretched human resources, and the COVID-19 pandemic in a rural setting in Namibia that can also be applied to other countries with similar challenges.

## MATERIALS AND METHODS

### Setting, structure, and funding.

Kavango East and Kavango West were selected for this program as they are Namibia’s most malarious regions, accounting for 47% of malaria cases and 40% of malaria deaths from January to June 2020 (J. Hamunyela, personal communication). These regions are characterized by low levels of vector control coverage, mobile and migrant populations, and some of Namibia’s lowest malaria reporting rates.^15^^,^^16^ Kavango East and Kavango West have separate political administrations of elected officials—namely, Regional Councils and Regional AIDS Coordinating Committees (RACOC)—but their health programs are managed by a shared Regional Management Team and are supported by four district coordinating committees.

Our 2-year program from June 2019 to July 2021 involved a series of facilitated group workshops and meetings accompanied by individual coaching and mentoring of health teams to strengthen capacity and provide opportunities for application of newly acquired skills. Using participatory methods, the program engaged multiple cadres from national, regional, district, facility, and community levels, within and beyond the malaria program (Table 1). To initiate the engagements, we convened an inception meeting for each approach where diverse perspectives from across the health system were represented in a collective exercise to identify and prioritize key challenges facing malaria program implementers. Cross-disciplinary teams prioritized challenges to focus on and developed strategies and action plans to address them over a 2-year period. We used qualitative and quantitative evaluation tools to monitor progress, adapt strategies and action plans in response to evolving circumstances and priorities, and assess the overall impact of the program.

**Table 1:** Roles and affiliations of participants in each capacity building approach of the joint program

	LEAD approach	Both	MBA approach
International	UCSF Program Coordinator, High Risk Population Surveillance Specialist, OD and leadership experts from South Africa, Zimbabwe, and University of the West of England		UCSF Advocacy Consultant; African Leaders Malaria Alliance; Elimination Eight Initiative; J.C. Flowers Foundation; Clinton Health Access Initiative
National	Quality Program Manager	National Malaria Program Officer	National Malaria Program Director, Directorate of Special Programs Resource Mobilisation and Development Coordinator, NACDO, UNAM, Society for Family Health Namibia
Regional	WHO Malaria Stopper	Regional Director, Chief Medical Officer, Chief Health Program Administrator, Chief Environmental Health Officer, Regional Surveillance Officer, Regional Clinical Malaria Mentor, Regional Malaria Coordinator, Regional Council representatives, Nurse Manager	Chief Medical Officer, Senior Accountant, Chief Immigration Officer, Senior Education Officer, Senior Social Worker, Rural Water & Sanitation Officer, Chief Community Liaison Officer: Gender, Information Officer
District	Health Information Systems Officer, Senior Medical Officer, Environmental Health Officer, Laborer, Faith-based organization representative	Senior Registered Nurse, Primary Health Care Supervisors, Registered Nurse, Malaria Clinical Mentors	
Community	Church representative, Constituency Council representatives, NGO representatives	Community health worker	Tribal Authorities representatives, DAPP Namibia, Teacher, Pastor, Namibia Red Cross Society, Police Officer

DAPP = Development Aid from People to People; LEAD = Leadership, Engagement, and Accountability for Improved Delivery of Services; MBA = Malaria Budget Advocacy; NACDO = Namibia Anglican Community Development Organization; NGO = nongovernmental organization; OD = organization development; UCSF = University of California–San Francisco; UNAM = University of Namibia.

### Approach 1: The LEAD Framework.

To resolve district health team management and operational barriers and strengthen leadership, we used the LEAD Framework from June 2019 to November 2020.^12^ In brief, external experts provided technical support in malaria elimination, quality improvement, organization development, and change leadership to improve programmatic effectiveness. Quality improvement and organization development are complementary in employing methods of team-based problem-solving and fostering ownership, responsibility, and accountability in the change process, with a significant emphasis on the skilled facilitation of these processes.^17^ Quality improvement, with an emphasis on measurement cycles, was informed by successes from prior HIV work and built on established capacity in Namibia.^18^

Cross-disciplinary Task Teams, consisting of members from the region, district, and community, convened quarterly throughout the program period to develop action plans to address the prioritized operational challenges. These meetings were an avenue for coaching, peer learning, sharing of best practices, and capacity-building on quality improvement and organization development and were professionally facilitated in-person or online. Between meetings, Task Team members implemented the action plans, including collecting data for monitoring and evaluation and supporting frontline staff to address challenges. At a final closeout workshop, Task Team members reported back on progress made, highlighted achievements, and proposed strategies to address unresolved and new challenges. This workshop and some of the later Task Team meetings were planned, designed, and facilitated by 12 of the Task Team members and staff from the national Ministry of Health and Social Services (MoHSS) under supervision from the external professional facilitators. This handover of the facilitation role was planned to ensure continued capability in this approach and was formalized through a certified training program. All 12 healthcare professionals successfully completed a postgraduate certificate in Professional Practice in Change Leadership (PPCL) that was delivered using blended learning (i.e., a combination of both virtual and in-person teaching) by faculty from the authors’ institutes. The training included the completion of individual projects, many of which focused on management challenges in the Task Team action plans, and the demonstration of skills to facilitate Task Team meetings and the closeout workshop.

### Approach 2: The MBA Framework.

**Figure 1.**
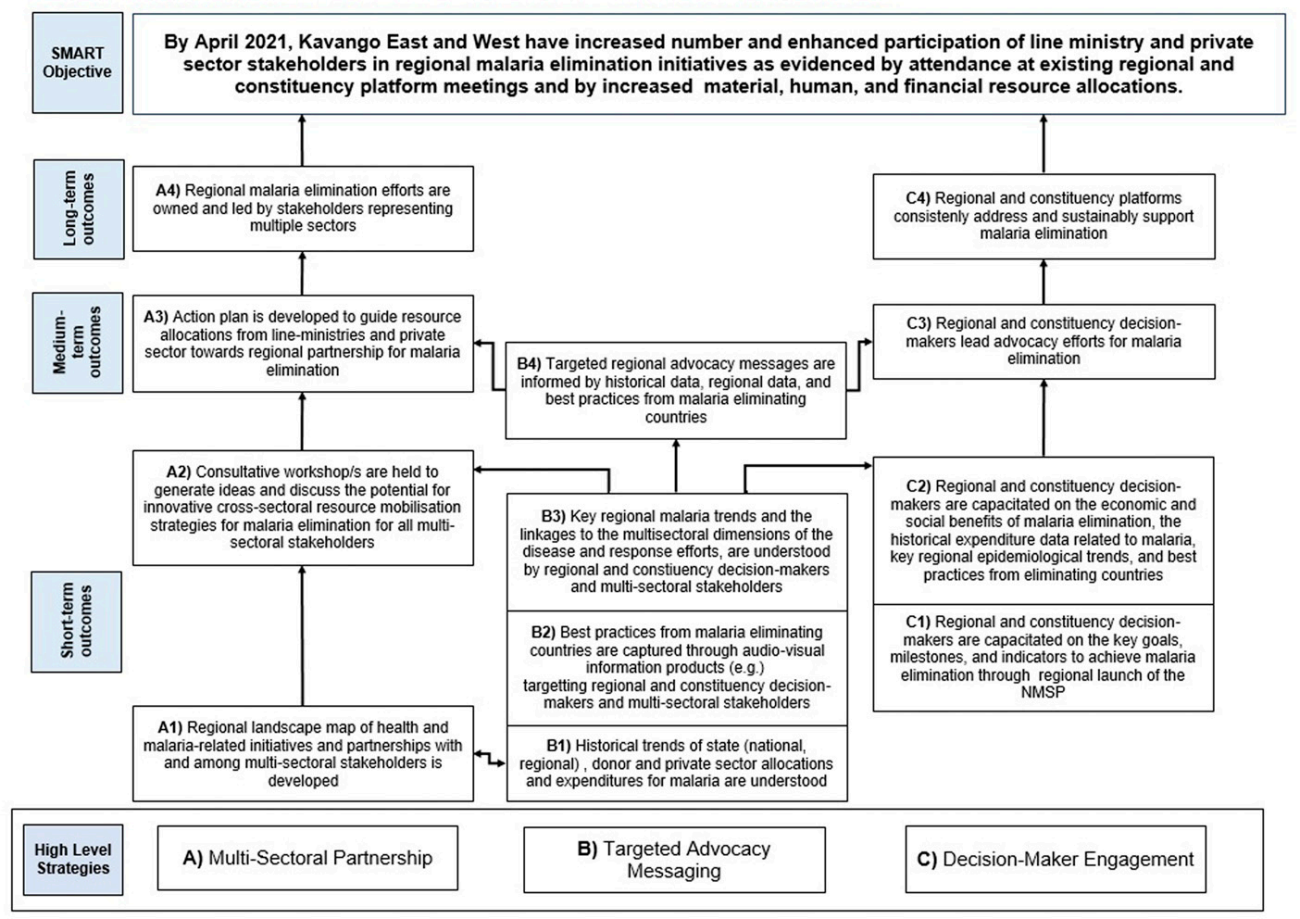
Kavango East and Kavango West Theory of Change (malaria budget advocacy approach). This figure appears in color at www.ajtmh.org.

To mobilize regional political commitment and domestic resources, we implemented the MBA Framework from August 2019 to July 2021.^13^ Participants engaged in this approach included health workers, local political officials, and finance managers. At the inception workshop, external experts oriented participants to budget advocacy and introduced the theory of change as a participatory tool to visualize how a strategy will logically achieve the intended impact—in this case, to strengthen subnational political commitment and resources for malaria. First, participants identified an advocacy objective that the group agreed they wanted to achieve by the end of the program period. Then, opportunities to influence policy, budget processes, and relevant decision-makers were identified through stakeholder and resource mapping activities. Facilitators and participants cocreated a theory of change (Figure 1) that mapped key outcomes and impact pathways. The theory of change served as a roadmap for action and a built-in monitoring and evaluation mechanism for tracking progress along the way.

To strengthen skills for implementation of the theory of change, budget monitoring, tracking, and advocacy experts from CEGAA administered a baseline capabilities assessment, delivered a week-long training with tailored curriculum, and adapted the curriculum into an online training series. CEGAA provided ongoing coaching and technical assistance throughout the project period, including conducting a comprehensive historical budget analysis. One Regional Health Directorate member from each region was identified as the regional focal point for advocacy implementation activities. Technical assistance, from CEGAA and a Namibia-based advocacy consultant, supported regional focal points in convening key decision-makers, generating advocacy evidence and materials, and ensuring linkages between district, regional, and national agendas.

## RESULTS

The program’s goals of resolving operational challenges, strengthening leadership, and mobilizing subnational political commitment and domestic resources for malaria elimination were met. Representative participant feedback on lessons and overall impact, outlined in Table 2, indicated that the program improved teamwork, communication, and problem-solving; increased support from the national level and engagement from the community in planning malaria activities; strengthened understanding of budget monitoring and advocacy skills; and fostered collaboration between regional political leadership and the Regional Health Directorate.

**Table 2:** Representative participant feedback from workshops

Capacity strengthening approach	Individual feedback on lessons and overall impact
LEAD	“Malaria activities are now part of the operational plans of the Kavango East Regional Council.”
	“Engage all stakeholders for a better outcome.”
	“Teamwork is crucial in order to combine resources and fight/eliminate malaria as well as creating awareness of programmes/activities to all corners of the region.”
	“The approach has informed other interventions and has demonstrated relevance, impact and effectiveness [in its early results.] It will be key to conduct further evaluation later (i.e., one year from now) to assess the sustainability and overall impact.”
	“The experience was an eye opening journey; [it] changed the way we see things.”
MBA	“The budgeting aspect has been very informative and helpful for me as a professional in public health, the prioritization process of needs for accessing the budget process has been insightful. Even just to understand that advocacy is such a diverse field and we had many eye-opening moments as a team.”
	“The regional advocacy strategy made the connection to the Regional Council for integrating malaria as a permanent feature at RACOC meetings.”
	“Without the advocacy strategy and this project [MBA] there would not be a working collaboration between the Regional Council and MoHSS in the region for malaria, I would say the working relationship moved from 20% to 95% because of the MBA initiative.”
	“I have seen a big impact on stakeholder engagement since MBA started, even just for the Regional Health Directorate to reach decentralized stakeholders and leaders in the constituencies and they are now more open to malaria messages.”
	“For me, the CEGAA training was great. It allowed me to understand the bigger picture of the budgeting process, and how me as a media practitioner can actually fit in, in terms of the messaging, how to construct or compile our messages accordingly. And in terms of budget advocacy, how we can advocate for our priorities to be considered.”
	“I learnt approaches on how to advocate for malaria. In malaria elimination, without finances there is nothing that we can do. So, we were taught how to prioritize. We were taught how to negotiate, which made it easier for me as a provincial director to advocate for political will.”

CEGAA = Center for Economic Governance and Accountability in Africa; LEAD = Leadership, Engagement, and Accountability for Improved Delivery of Services; MBA = Malaria Budget Advocacy; MoHSS = Ministry of Health and Social Services; RACOC = Regional AIDS Coordinating Committee.

### LEAD.

Significant progress was made on five out of six operational challenges in the Task Teams’ action plans, as shown in Table 3. During the period of intervention from June 2019 to November 2020, this included the following results: an observed 40% increase in malaria case reporting (both districts: 60–100%), a 32% increase in reporting and tracing of imported malaria cases (Nankudu: 41–79%; Rundu: 20–45%), and a 10% increase in malaria case management (Nankudu: 89–100%; Rundu: 89–98%). The limited availability of vehicles and insecticide continued to impact IRS coverage rates throughout the program, as they were challenges that needed to be elevated to the national level. Table 3 also summarizes the Task Teams’ action plans, including the challenges and solutions. In addition to the outcomes in Table 3, the formation of the district Task Teams had positive spillover effects, stimulating action at the national level and expansion to other districts. A direct line of communication was created between the Task Team and the NVDCP to address shortages of malaria drugs, insecticide, vehicles, and human resources. Due to Task Team advocacy, the national program provided six vehicles for malaria activities, gave approval to hire additional staff, and provided funding for 10 additional days of active case detection from what had been budgeted.

**Table 3:** Task Team action plans for Leadership, Engagement, and Accountability for Improved Delivery of Services

Challenge	Solution	Baseline	Endline	Notes
**Resolved process measures**				
Treatment of confirmed malaria cases according to guidelines	Conduct in-service training at all health facilities, weekly monitoring of drug stocks, diagnosis, and treatment, provide phone and in-person support	Nankudu: 89% Rundu: 89%	Nankudu: 100% (2,778/2,778 cases) Rundu: 98% (1,326/1,353 cases)	
Late and incomplete malaria reporting from health facilities	Use of any form of communication to ensure malaria case reporting within 24 hours, fix broken tablets, design tool to measure timeliness/ completeness	Nankudu: 60% Rundu: 60%	Nankudu: 100% Rundu: 100%	
**Resolved structural measures**				
Community engagement for malaria activities	Conduct health education at health facilities and in communities, mobilize villages to prepare them for spraying of homes with insecticide	Nankudu: 80% IRS coverage Rundu: 88% IRS coverage	Nankudu: 46% (71/155 villages sprayed) Rundu: 55%	Low coverage due to chemical stockouts, vehicle shortage in both districts. Nankudu: 74% (115/155) of villages were mobilized Rundu: 98% (24/25) of health education sessions conducted
Cross border collaboration with Angola	Shared imported cases with Angola counterpart via WhatsApp	Nankudu: 41% Rundu: 20%	Nankudu: 79% (55/70) traced by Angola Rundu: 45% (41/91)	Rundu: Rapid case notification forms not shared on time from facility level
Lack of stakeholder engagement in malaria	Conduct regular meetings with stakeholders	Nankudu: 4 meetings Rundu: 4 meetings	Nankudu: 5 meetings conducted due to meeting restrictions, shared data and coordinated joint work Rundu: 4 meetings	Malaria included as an agenda item for the Constituency & Regional AIDS Coordinating Committees Rundu: 5 new stakeholders engaged
**Unresolved structural measures**				
Availability of vehicles for malaria surveillance and vector control	Combine trips for active case detection, indoor residual spraying, larviciding, request vehicles from the region or other stakeholders	Nankudu: 8 vehicles; Rundu: 8 vehicles	Nankudu: 6 vehicles; Rundu: 9 vehicles	Difficult to combine trips because active case detection team consists of 5 members, leaving no room for others; 93% (163/175) of water bodies treated with larvicide, need to elevate issue from the regional to national level

### MBA.

The budget advocacy objective set in the theory of change (Figure 1) was to increase and broaden participation in regional malaria elimination efforts through three interim outcomes: 1) the establishment of multi-sectoral Malaria Elimination Task Forces (METFs) within RACOCs; 2) the integration of malaria into the regional political agenda to pave the way for mobilization of resources; and 3) increased use of financial evidence in budget advocacy.

A regional METF was established within each region’s RACOC that included leaders from governmental (health and other line ministries), nongovernmental, private, faith-based, and academic sectors. The METFs in both regions successfully integrated malaria activities into the standing agendas of their Regional Council for the first time. Since their establishment in late 2020, the METFs have already secured funding for transport and meetings for malaria response. The METFs plan to advocate for additional domestic resources in the next fiscal year budget cycle by securing malaria’s inclusion in the Regional Council’s operational plan and budget. Forty-six multisectoral stakeholders were trained by CEGAA on budget monitoring, expenditure tracking, and advocacy skills to strengthen effectiveness of region-led budget advocacy through increased use of financial evidence. Participants applied their skills and knowledge in establishing the multisectoral METFs, using advocacy to generate political buy-in to prioritize resource mobilization for malaria, and securing in-kind resources for malaria elimination from new line ministries and private sector stakeholders.

### Increased integration and community engagement for malaria.

Both approaches aimed to expand the coalition of subnational, multisectoral stakeholders who are active in malaria elimination efforts. This focus culminated in successfully integrating malaria activities into the RACOC standing agenda and increasing community acceptance of IRS through tailored district-level sensitization events. The strategy of increasing decision-maker engagement overlapped with the community engagement operational challenge (Table 3). The Regional Health Directorate and Tribal Authorities cohosted four district-level community engagement events across both regions to increase community acceptance of IRS through involvement of religious leaders, private sector entities, constituencies, and other stakeholders. Postevent reports indicated the districts that hosted the IRS campaign launch events saw reductions in refusals of IRS in 2020 compared with 2019 and increased buy-in of traditional leaders, who carry great community influence.

### Impact of COVID-19.

The emergence of COVID-19 in Namibia resulted in disruptions to planned malaria activities and our program. Emergency response regulations stretched human resources across the MoHSS, diverting their attention to COVID-19 activities, restricted travel and meeting sizes, disrupted the national budget cycle, blocked importation of insecticide and LLINs, narrowed the scope of the program strategies and action plans, and resulted in a shift to a hybrid model of in-person and virtual support. Despite this adaptation, participants indicated that the quality of the interactions between the virtual facilitators and in-person participants was not compromised by the substitution of virtual for in-person facilitation. Additionally, these interactions helped the district and regional teams through challenging times by showing how organization development facilitation can aid rapid and effective adaptation to emerging circumstances and how the LEAD Framework and tools can readily be applied in these circumstances.

### Program costs.

Both the LEAD and MBA approaches required the use of consultants, technical assistance providers, rental of meeting venues for convenings, transportation and travel, and payment of per diems for meeting participants amounting to USD ∼36,000 per district per year for the LEAD approach and USD ∼25,000 per region per year for the MBA approach.

### Program sustainability.

Program participants have reported applying and integrating newly acquired and strengthened skills to their daily work. Both the Regional Health Directorate and district health teams will continue resolving prioritized management and advocacy challenges identified during the inception workshops across all health areas. The Task Teams began meeting on their own and continue to meet without external support, having incorporated their activities into existing NVDCP workplans. Minimal resources are needed for these teams to meet, coordinate, and resolve issues in relation to the impact that they can have in identifying efficiencies, improving effectiveness, and increasing political and financial commitment.

Establishment of the regional METFs enables the integration of malaria into the Regional Council operational plans and absorption of district Task Team members, providing a pathway for accessing resources for malaria activities from the Regional Council budget and an avenue for the regions to be informed of operational challenges at the district level. By nature of being hosted and institutionalized within an existing political platform, the METFs will convene on a quarterly schedule without external financial or technical support.

Due to interest expressed by the two other districts in Kavango East and Kavango West that were not part of the original LEAD approach, stakeholders were oriented to these methods. In addition to Kavango East and West, four other regions in northern Namibia were trained in malaria budget advocacy methods and two others adopted the METF model. Further expansion of the LEAD approach can be led by the 12 PPCL-certified graduates in Namibia. The authors’ institute/CEGAA MBA training curriculum was adapted UCSF an open-access web-based series and resource mobilization advocacy priorities were integrated into Namibia’s recently updated 2020–2025 Malaria Communications and Advocacy Strategy.^19^

## DISCUSSION

In partnership with Namibia’s MoHSS, we set out to strengthen subnational capacity to resolve operational challenges, increase political commitment and community engagement, and mobilize domestic resources for malaria elimination. Political support from the NVDCP played an important role in this achievement by the end of the implementation period. Because of the timing of budget cycles, the METFs have not yet observed increases in regional budgets resulting from their advocacy. The establishment of the METFs has diversified engagement of multisectoral leadership, embedded a malaria agenda within political platforms at regional level, and increased visibility and political commitment to malaria elimination. Another limitation of this analysis is that it is not possible to unambiguously attribute all outcomes to our program alone. We have relied on the triangulated assessment of the resolved process and structural measures of the key actors, who are in the best position to weigh their influence alongside parallel activities in the Namibian health services. Additionally, there were no other additional investments to supplement the government resources that could have contributed to the outcomes.

The program was catalytic, and each approach benefitted the other. The Task Teams found that political decision-makers were more receptive to their appeals because of elevated awareness and prioritization of malaria, and the operational challenges within the Task Team action plans provided evidence for regional METFs to use in their “asks” for district-level programmatic needs such as vehicles, drivers, fuel, and radio spots. Our work also stimulated other partners to devote funds to raising awareness about malaria among political leaders in the neighboring district of Andara.

Some lessons for future application of our program include that participatory evaluation does not always yield metrics that accurately capture progress unless teams are provided continual feedback and guidance. This underscores the utility of providing virtual or in-person technical and measurement support. We also learned that it would have been more efficient to identify a shared focal point for the LEAD and MBA approaches at the district level to ensure alignment and synergy.

Future iterations of the program could involve application of budget management skills toward financial planning and mobilizing subnational financial resources to address ongoing management challenges, such as procuring and fixing vehicles, a structural problem prevalent throughout Namibia’s health system that inhibits the achievement of processes and outcomes. A combined leadership, management, and advocacy program planned and implemented jointly from the start would reduce the number of inception and close-out workshops and the implementation budget.

This article aimed to demonstrate the utility and benefits of applying the “soft” skills of improved teamwork, communication, group decision-making, prioritization, advocacy, and budget management at the subnational level to improve commonly stated challenges to disease control. These include multistakeholder engagement, health system operational challenges of limited personnel and transport, and cross-disease program efficiencies. In this case, we show the relevance of skill-building for malaria elimination. Although the outcomes are self-reported and there is no control group to establish causality, we show that such programs can be instituted at little cost and are likely to result in improved health system performance.

## CONCLUSION

Subnational health teams are increasingly responsible for operationalizing and financing the local malaria response as the disease burden falls, health systems decentralize, and donor assistance declines. The combined implementation of two complementary, participatory approaches (LEAD and MBA Frameworks) in Namibia strengthened subnational leadership, management, and advocacy skills, leading to broad-ranging outcomes that included the resolution of operational challenges, improvements to the quality of malaria service delivery, greater political commitment, and the ability to mobilize domestic resources for malaria elimination. We encourage other countries to consider employing this program to strengthen the health system, improve planning, budgeting, problem-solving, and decision-making capacity across a range of stakeholders, and promote resilience and sustainability.
